# Tree shrew (*Tupaia belangeri chinensis*), a novel non-obese animal model of non-alcoholic fatty liver disease

**DOI:** 10.1242/bio.020875

**Published:** 2016-09-22

**Authors:** Linqiang Zhang, Xiaoyun Wu, Shasha Liao, Yunhai Li, Zhiguo Zhang, Qing Chang, Ruyue Xiao, Bin Liang

**Affiliations:** 1 Key Laboratory of Animal Models and Human Disease Mechanisms of Chinese Academy of Science & Yunnan province, Kunming Institute of Zoology, Chinese Academy of Sciences, Kunming, Yunnan 650223, China; 2 Kunming College of Life Science, University of Chinese Academy of Sciences, Kunming, Yunnan 650204, China; 3 Key Laboratory of Puer Tea Science, Ministry of Education, Yunnan Agricultural University, Kunming, Yunnan 650201, China; 4 School of Life Sciences, Anhui University, Hefei, Anhui 230601, China; 5 Pharmaceutical College, Heilongjiang University of Chinese Medicine, Harbin 150040, China

**Keywords:** Non-alcoholic fatty liver disease (NAFLD), Tree shrew (*Tupaia belangeri chinensis*), High energy diet, Animal model, Non-obese fatty liver

## Abstract

Non-alcoholic fatty liver disease (NAFLD) is becoming a severe public health problem that is affecting a large proportion of the world population. Generally, NAFLD in patients is usually accompanied by obesity, hyperglycemia, insulin resistance (IR) and type 2 diabetes (T2D), for which numerous animal models have been generated in order to explore the pathogenesis and therapies of NAFLD. On the contrary, quite a number of NAFLD subjects, especially in Asian regions, are non-obese and non-diabetic; however, few animal models are available for the research of non-obese NAFLD. Here, four approaches (here called approach 1 to 4) corresponding to the variable compositions of diets were used to treat tree shrews (*Tupaia belangeri chinensis*), which have a closer evolutionary relationship to primates than rodents. Analysis of plasma biochemical parameters, hepatic histology, and the expression of hepatic lipid metabolic genes revealed that all four approaches led to hepatic lipid accumulation, liver injury and hypercholesterolemia, but had no effect on body weight and adipose tissue generation, or glycemia. Hepatic gene expression in tree shrews treated by approach 4 might suggest a different or non-canonical pathway leading to hepatic steatosis. In conclusion, the tree shrew displays hepatic steatosis and dyslipidemia, but remains non-obese and non-diabetic under high energy diets, which suggests that the tree shrew may be useful as a novel animal model for the research of human non-obese NAFLD.

## Abstract (precis)

**Summary:** This study suggests that the tree shrew (*Tupaia belangeri chinensis*) may be useful as a novel animal model for the research of human non-obese non-alcoholic fatty liver disease (NAFLD).

## INTRODUCTION

Non-alcoholic fatty liver disease (NAFLD), is a clinicopathological liver disorder characterized by macrovesicular hepatic lipids accumulation and occurs in patients who consume little or even no alcohol (Angulo, 2002; Mulhall et al., 2002; Neuschwander-Tetri and Caldwell, 2003; Sanyal, 2002). Clinically, the risk factors and the output of hepatic histopathology and pathophysiology among human NAFLD are numerous and complex, encompassing a spectrum of liver damage varying from simple steatosis, in which more than 5% of hepatocytes present lipid accumulation in the form of lipid droplets (Szczepaniak et al., 2005), to non-alcoholic steatohepatitis (NASH), and eventually fibrosis and cirrhosis (Angulo and Lindor, 2002; Matteoni et al., 1999). Generally, NAFLD subjects usually display other associated metabolic symptoms including obesity, dyslipidemia, insulin resistance and type 2 diabetes (Adams and Lindor, 2007; Angulo and Lindor, 2002; Clark et al., 2002; Targher and Arcaro, 2007). However, NAFLD also occurs in patients who show non-obese and non-diabetic symptoms (Chow et al., 2007; Goh et al., 2016; Omagari et al., 2002), and a high percentage (15–21%) of Asia-Pacific NAFLD subjects have been found to be non-obese.

Due to the difficulty of clinical studies, animal models are absolutely necessary to explore the pathogenesis and therapies of NAFLD. To date, numerous animal models, especially rodents, have been generated by different approaches for the research of human NAFLD, including spontaneously genetic mutation models such as *ob/ob* mouse (Mayer et al., 1951), *db/db* mouse (Hummel et al., 1966), Zucker rats (Yang et al., 1997), as well as dietary or pharmacological manipulated models. For example, high energy diets (high fat diet, high cholesterol diet, or high carbohydrate diet) induced NAFLD in rodents (Takahashi et al., 2012), rabbits (Fu et al., 2009; Ogawa et al., 2010), opossums (Chan et al., 2012), and Ossabaw pigs (Lee et al., 2009). Regardless of the types of animal models used, the common characteristics are hepatic steatosis accompanied by obesity, dyslipidemia, hyperglycemia, insulin resistance and hyperinsulinemia (Anstee and Goldin, 2006; Ibrahim et al., 2015; Kucera and Cervinkova, 2014; Sanches et al., 2015), which largely reflect the histopathology and pathophysiology of human NAFLD in obese and/or diabetic patients. Therefore, these animal models are extremely valuable to study the pathogenesis and therapies of human NAFLD in obese and/or diabetic patients. There are very few animal models that have been reported to be useful for the research of non-obese and non-diabetic human NAFLD. Hence, developing new animal models of non-obese and non-diabetic NAFLD appears to be both urgent and essential.

The tree shrew (*Tupaia belangeri chinensis*) is a close relative of primates in terms of evolution (Fan et al., 2013), and has been used in biological research especially for hepatitis B virus (HBV) and C virus (HCV) research for decades (Su, 1987; Walter et al., 1996; Yan et al., 1996). Previously, we reported that the relationships between body weight, fasting blood glucose concentration, sex and age in tree shrews are similar to that of human beings. Additionally, we recently showed that a high fat diet combined with cholesterol successfully induced liver steatosis to inflammation and fibrosis progressively within 10 weeks, but induced no change in body weight (Zhang et al., 2015b), which was distinct from the cases of mice and Ossabaw pigs. This raised the question of whether or not the tree shrew could be an alternative animal model specific for the research of non-obese and non-diabetic human NAFLD. Therefore, we applied different types of high-energy diets including high fat or high sucrose diets to treat tree shrews. Remarkably, all these diets successfully induced liver steatosis, but did not affect body weight, suggesting that the tree shrew is a novel animal model of human non-obese NAFLD.

## RESULTS

### High-energy diets caused liver injury in tree shrew

**Fig. 1.**
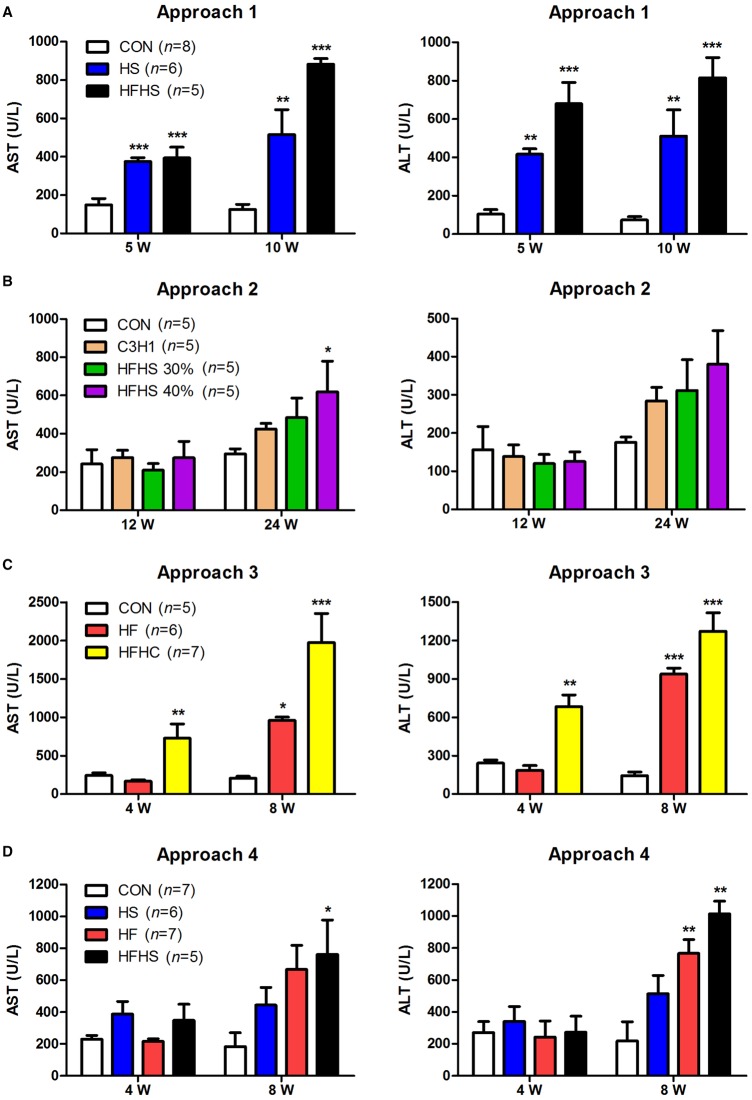
**The plasma levels of AST and ALT.** Approach 1 (A), 2 (B), 3 (C), and 4 (D). Data were presented as mean±s.e.m. Biological repeats (*n*) are shown within the figure. Significant difference between the CON diet group and an indicated diet group at the same time point: *P<0.05, **P<0.01, ***P<0.001 (ANOVA). CON, control diet; HS, high sucrose diet; HF, high fat diet; HFHS, high fat and high sucrose diet; HFHC, high fat and high cholesterol diet.

As markers of liver necroinflammation, plasma aspartate aminotransferase (AST) and alanine aminotransferase (ALT) levels have been widely used to indicate the degree of liver injury. In approach 1 (including high sucrose diet, and high fat and high sucrose diet; Table S1 and S2), the plasma levels of AST and ALT were normal in the control (CON) group, but increased gradually and significantly in both the high sucrose (HS), and the high fat and high sucrose (HFHS) groups at 5 weeks and 10 weeks (Fig. 1A). In approach 2 (including various proportions of high fat and high sucrose diet; Table S1 and S2), the plasma levels of AST and ALT were mostly normal throughout the experimental duration (24 weeks) even though the level of AST was slightly higher in the HFHS group than the CON group at 24 weeks (Fig. 1B). Astoundingly, in approach 3 (including high fat diet, and high fat and high cholesterol diet; Table S1 and S2), a great increase of plasma AST and ALT levels was seen in the high fat and high cholesterol (HFHC) group from 4 weeks to 8 weeks, which was ∼3× higher at 4 weeks and 9× higher at 8 weeks than the CON group (Fig. 1C). In approach 4 (similar to the approach 1, but fed animals high energy diets every other day; Table S1 and S2), the plasma levels of AST and ALT showed no difference among four groups at 4 weeks, but significantly increased in the HFHS group at 8 weeks (Fig. 1D). Altogether, high-energy diets tested in approaches 1, 3 and 4, but not in approach 2, caused liver injury in tree shrew.

### Different diet approaches changed blood lipid profiles in tree shrew

**Fig. 2.**
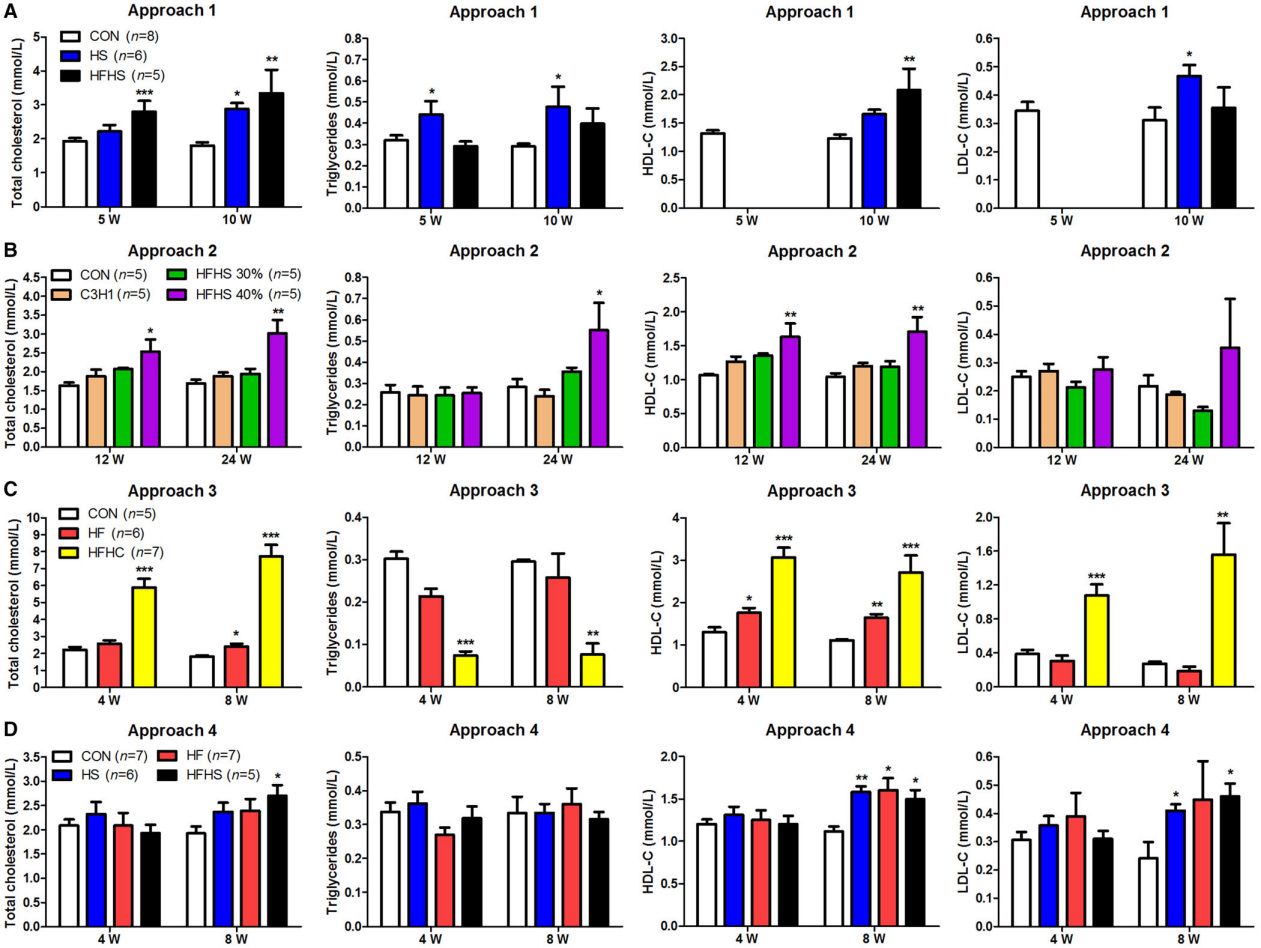
**Blood lipid profiles**. Approach 1 (A), 2 (B), 3 (C), and 4 (D). The HDL-c and LDL-c levels of HS and HFHS group in approach 1 at 5 weeks were not detected. Data were presented as mean±s.e.m. Biological repeats (*n*) are shown within the figure. Significant difference between the CON diet group and an indicated diet group at the same time point: *P<0.05, **P<0.01, ***P<0.001 (ANOVA). CON, control diet; HS, high sucrose diet; HF, high fat diet; HFHS, high fat and high sucrose diet; HFHC, high fat and high cholesterol diet.

NAFLD patients often display dyslipidemia. Consistently, high-energy diets often caused dyslipidemia in animal models of NAFLD (Table S3). In order to investigate whether or not high-energy diets lead to dyslipidemia in tree shrews, we tested four common indicators of dyslipidemia: triglycerides (TG), total cholesterol (TC), low-density lipoprotein cholesterol (LDL-c) and high-density lipoprotein cholesterol (HDL-c). In approach 1, the plasma TC levels were elevated gradually and significantly in both the HS and the HFHS group. More specifically, both the plasma TG level and the LDL-c level increased significantly in the HS group but not in the HFHS group, whereas the change in the HDL-c level was the opposite (Fig. 2A). In approach 2, only in the HFHS (40%) diet group did the plasma levels of TC, TG and HDL-c in the tree shrew increase significantly (Fig. 2B). In approach 3, the HF diet significantly elevated the TC level at 8 weeks and the HDL-c level at both 4 weeks and 8 weeks (Fig. 2C). Remarkably, the HFHC diet greatly increased the levels of TC, HDL-c and LDL-c, and decreased the level of TG at both 4 weeks and 8 weeks (Fig. 2C), which was consistent with our previous report (Zhang et al., 2015b). Additionally, in approach 4, none of the four diets affected the TG level throughout the experimental duration, or the levels of TC, HDL-c and LDL-c at 4 weeks. Only the HFHS diet significantly increased the levels of TC, HDL-c and LDL-c (Fig. 2D). Taking all these lines of evidences together, different approaches with distinct diets can indeed cause dyslipidemia to various degrees within tree shrew.

### High-energy diets did not cause hyperglycemia

**Fig. 3.**
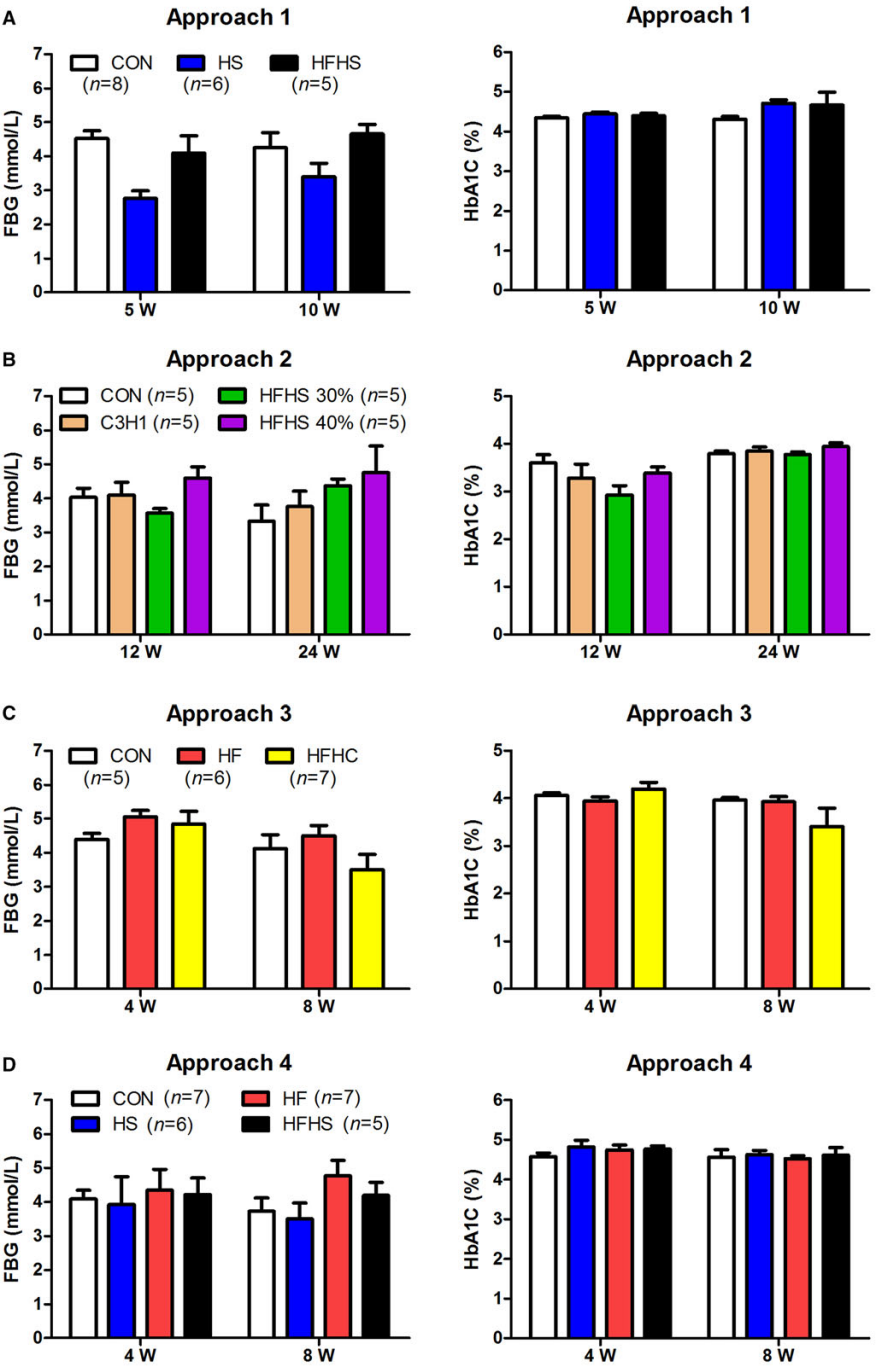
**The levels of fasting blood glucose (FBG) and glycated haemoglobin A1c (HbA1c).** Approach 1 (A), 2 (B), 3 (C), and 4 (D). Data were presented as mean±s.e.m. Biological repeats (*n*) are shown within the figure. CON, control diet; HS, high sucrose diet; HF, high fat diet; HFHS, high fat and high sucrose diet; HFHC, high fat and high cholesterol diet.

In all four approaches, the levels of fasting blood glucose (FBG) and glycated haemoglobin A1c (HbA1c) showed no significant change at any experimental time point when compared to the CON group, regardless of the HF (high fat), HFHC, HS, or HFHS diet groups (Fig. 3). Thus, the tree shrew displays no hyperglycemia under the induction of high-energy diets (Table S3).

### High-energy diets led to hepatic steatosis

**Fig. 4.**
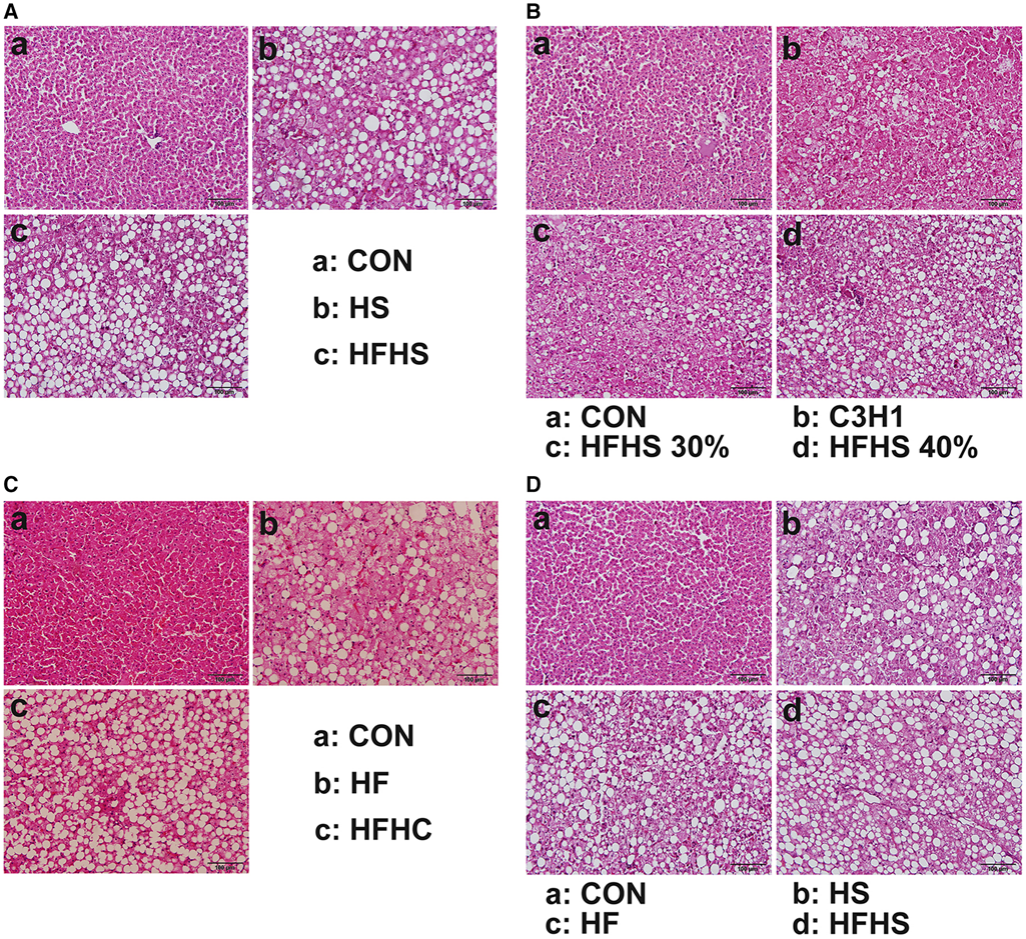
**Haematoxylin and eosin (H&E) staining of liver sections.** Approach 1 (A), 2 (B), 3 (C), and 4 (D). Scale bars: 100 μm. CON, control diet; HS, high sucrose diet; HF, high fat diet; HFHS, high fat and high sucrose diet; HFHC, high fat and high cholesterol diet.

Lipids accumulation in the liver is the most remarkable feature of NAFLD in numerous animal models as well as in human patients. As the gold-standard for reflecting hepatic steatosis, pathological section by haematoxylin and eosin (H&E) staining is often used to detect liver lipid accumulation in both clinical diagnosis (reviewed by Tannapfel et al., 2011) and animal research (He et al., 2013; Huang et al., 2015; Lee et al., 2015). Therefore, liver tissues from the end time-point of each approach were processed by histopathology (H&E staining) and the histological changes were visualized by microphotographs (Fig. 4). The liver sections from the control groups displayed normal hepatocytes, whereas the sections from all high-energy diet groups exhibited numerous huge pathological vacuoles in hepatocytes (Fig. 4), suggesting that lipid droplets accumulated in liver, and that these animals developed hepatic steatosis (Table S3).

### High-energy diets had no effect on adipose tissues

**Fig. 5.**
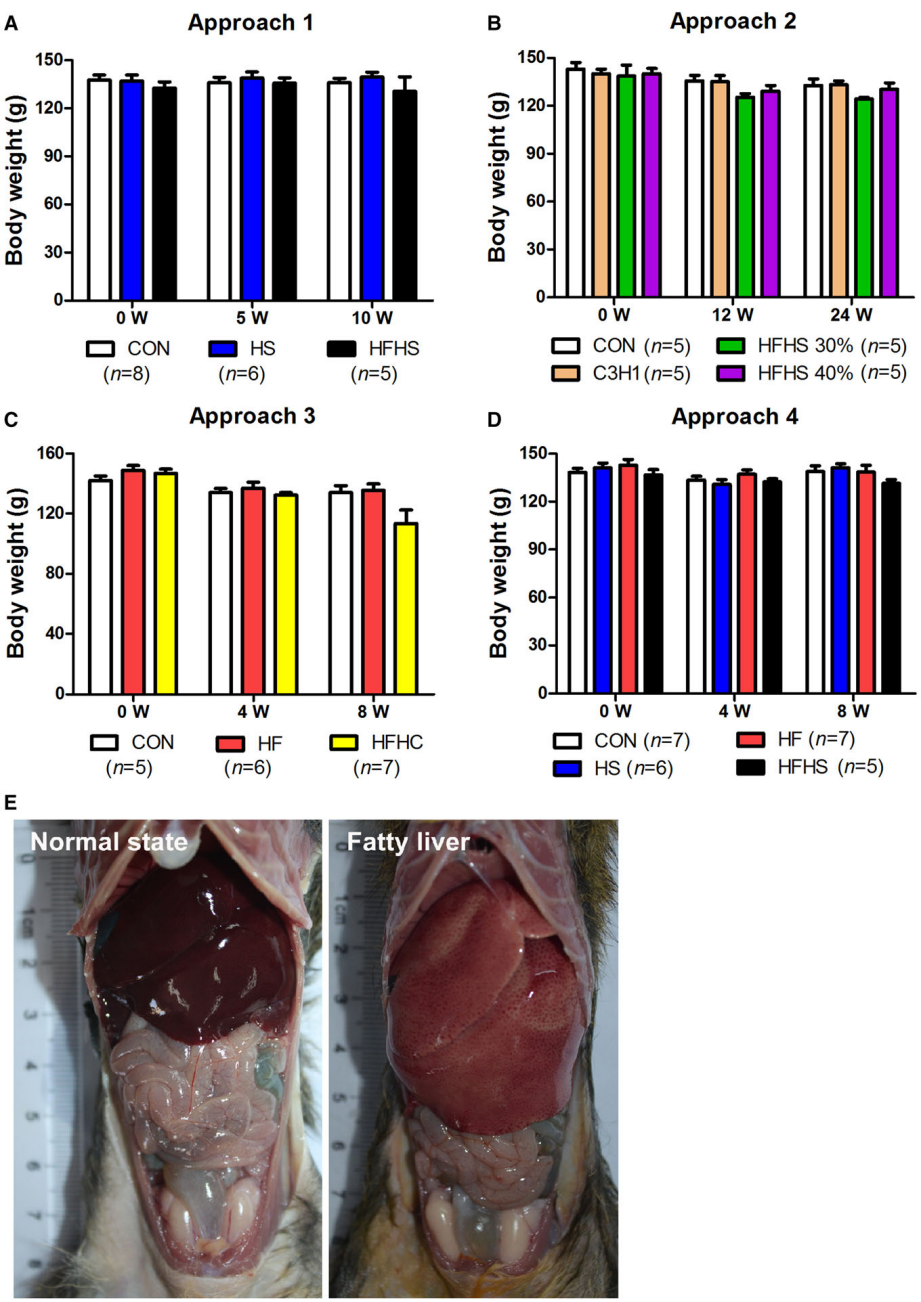
**The body weight and dissections of animals fed on CON diet and HFHC diet.** Body weight shown in approach 1 (A), 2 (B), 3 (C), and 4 (D). Data were presented as mean±s.e.m. Biological repeats (*n*) are shown within the figure. (E) Dissections of animals fed on CON diet (normal state) and HFHC diet (fatty liver) from approach 3. CON, control diet; HS, high sucrose diet; HF, high fat diet; HFHS, high fat and high sucrose diet; HFHC, high fat and high cholesterol diet.

High-energy diets generally enlarge adipose tissues leading to obesity in most experimental animals and humans. Interestingly, when compared with control groups, neither the HF diet, the HS diet, nor the HFHS diet in approach 1, 2 and 4 affected body weight during the experiment (Fig. 5A-D, Table S3). Although the high fat diet (HF) did not affect body weight during the experiment in approach 3, the high fat and high cholesterol (HFHC) diet eventually led to a slightly decreased body weight at the end of the experiment (8 weeks) (Fig. 5C, Table S3), which was consistent with our previous report (Zhang et al., 2015b). In addition, animal dissection showed that adipose tissues, including subcutaneous adipose tissues, visceral adipose tissues and epididymal adipose tissues, were apparently all absent except the enlarged and pale livers in high-energy diet-induced animals (Fig. 5E), even though there were no differences in food consumption among the groups within any approach (data not shown). Altogether, these results indicate that high-energy diets exclusively lead accumulation of fat in the liver, without accompanying obesity in tree shrew.

### High-energy diets impaired the expression of lipid metabolic genes

**Fig. 6.**
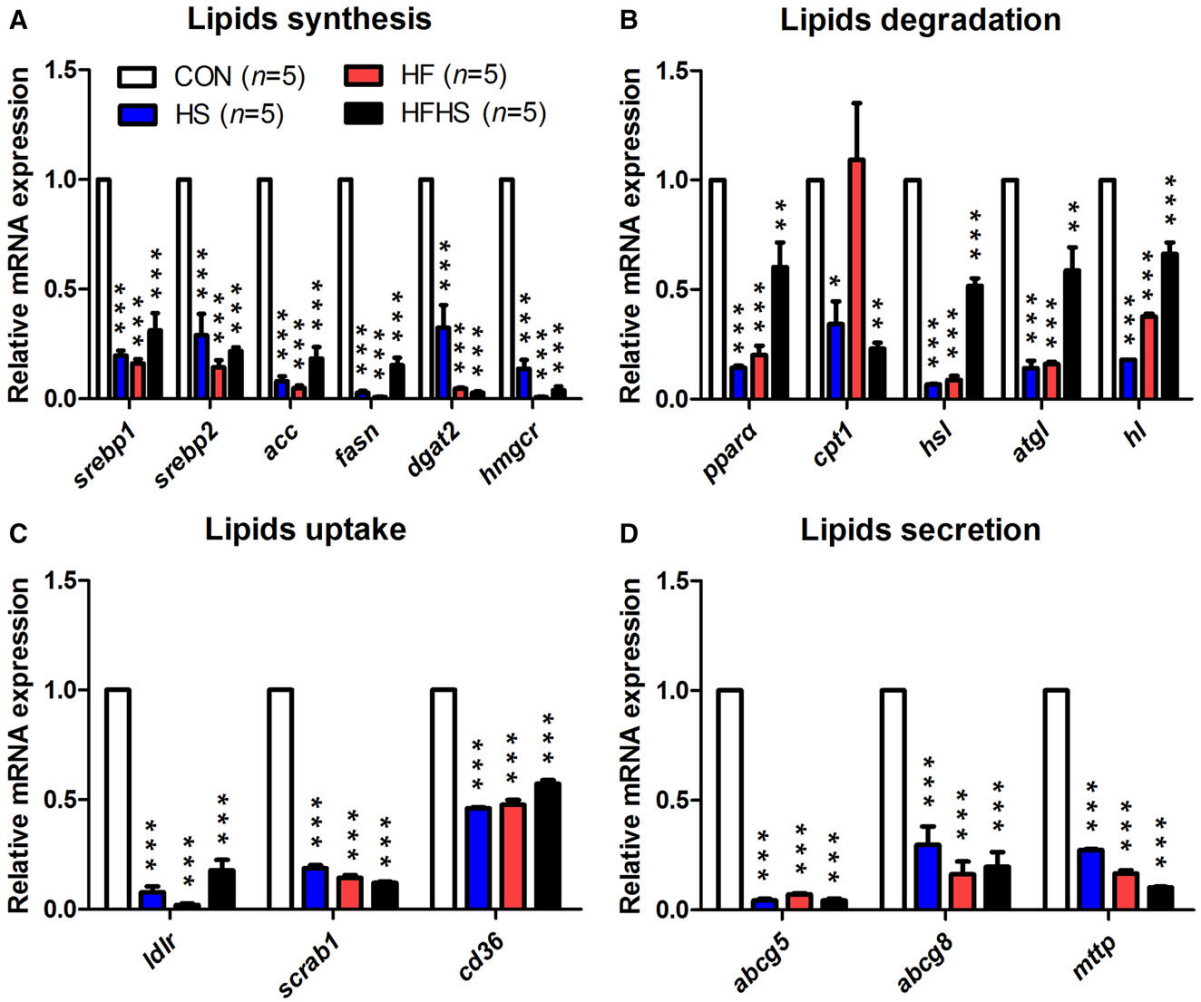
**The mRNA expression of lipid metabolic genes involved in lipids synthesis, degradation, uptake, and secretion.** Lipids synthesis (A), degradation (B), uptake (C), and secretion (D). Data were presented as mean±s.e.m. Biological repeats (*n*) are shown within the figure. Significant difference between the CON diet group and an indicated diet group at the same time point: *P<0.05, **P<0.01, ***P<0.001 (ANOVA). CON, control diet; HS, high sucrose diet; HF, high fat diet; HFHS, high fat and high sucrose diet; HFHC, high fat and high cholesterol diet.

In order to investigate the underlying mechanisms of lipid accumulation in the liver of tree shrews, the mRNA expression of some genes involved in lipid synthesis, degradation, uptake and secretion were tested by real-time quantitative PCR (QPCR) in approach 4. Interestingly, the expression of all 17 lipid metabolic genes was significantly down-regulated in the HF, HS, and HFHS groups compared to the control group (Fig. 6A-D), suggesting that high-energy diets do impair lipid metabolism in the liver of tree shrews.

## DISCUSSION

Excessive intake of energy is regarded as one of the crucial inducements of NAFLD; therefore, high-energy diets are commonly applied to generate animal models of NAFLD. In this study, four types of high-energy diets were applied, namely high sucrose diet (HS), high fat diet (HF), high fat and high sucrose diet (HFHS), as well as high fat and high cholesterol diet (HFHC). We used the combination of different diets in approaches 1 to 4 to treat tree shrews, and found that all high-energy diets lead to hepatic steatosis.

The majority of human NAFLD patients are associated with metabolic risk factors such as obesity, insulin resistance and type 2 diabetes, which have been extensively studied; however, concerns regarding non-obese human NAFLD are rising as it is not uncommon in Asian subjects, who often possess lower BMI cut-offs (Liu, 2012). Clinically, the waist circumference, total abdominal fat levels, and subcutaneous fat levels were significantly higher in obese NAFLD patients than in non-obese NAFLD patients who did not show obvious insulin resistance (Yasutake et al., 2009). The same can be found of the prevalence of type 2 diabetes, the plasma TG and HDL-c levels (Honda et al., 2016). The laboratory animals, mice and rats, have been extensively used for the research of human NAFLD since they generally display hepatic steatosis, obesity including increased body weight and white adipose tissue (WAT), hypertriglyceridemia, hypercholesterolemia and hyperglycemia when fed on high energy diets (Table S3). Consistent with human and rodent NAFLD, tree shrew NAFLD presented hepatic lipid accumulation, and with hypercholesterolemia on all four diets, as well as hypertriglyceridemia on the HS diet (Table S3). However, the tree shrews treated by these four approaches did not gain body weight (Fig. 5A-D), or epididymal or intra-abdominal adipose tissues (Fig. 5E), the levels of FBG and HbA1c did not increase (Fig. 3). Therefore, those lines of evidences indicate that unlike rodent models, the tree shrew model of NAFLD is non-obese and non-diabetic, which fundamentally mimics the principal symptoms of non-obese human NAFLD subjects.

In rodent models of NAFLD, once hit by excessive energy, the livers displayed a disorder of lipid metabolism which consequently led to lipid accumulation. In general, genes involved in lipid synthesis (*srebp1*, *srebp2*, *acc*, *fasn*, *dgat2* and *hmgcr*) and uptake (*ldlr*, *scrab1* and *cd36*) have up-regulated, while genes involved in lipid degradation (*pparα*, *cpt1*, *hsl*, *atgl* and *hl*) and secretion (*abcg5/8* and *mttp*) have down-regulated at mRNA or protein level (Ann et al., 2015; Gou et al., 2016; Hou et al., 2016; Kang et al., 2016; Komiya et al., 2016; Li et al., 2016; Liu et al., 2016; Ren et al., 2014; Shindo et al., 2010; Van Rooyen et al., 2011; Zhang et al., 2015a). Consistent with rodent models, the mRNA expression of genes involved in lipid degradation and secretion was decreased in tree shrews treated by approach 4 (Fig. 6B,D). However, surprisingly, both the mRNA expression of lipid synthesis and uptake pathway genes were decreased (Fig. 6A,C). Although the underlying mechanisms of lipid accumulation in tree shrew need to be further studied, the discrepancy of lipid gene expression between tree shrew and rodents somehow suggests that a new or non-canonical pathway may exist in tree shrews as the result of hepatic steatosis under excessive energy induction, which hopefully will provide new clues to investigate the pathogenesis of human non-obese NAFLD.

**Fig. 7.**
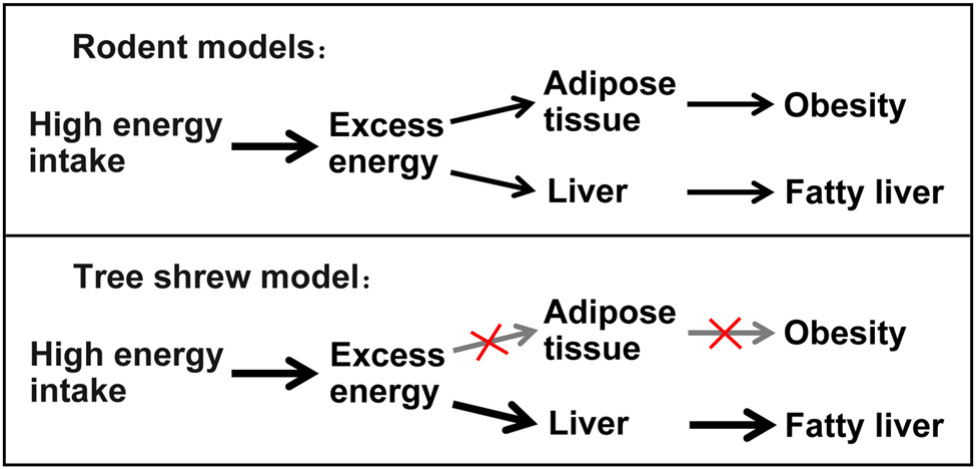
**A model of energy flow and distribution between rodent models and tree shrew model of NAFLD.** Once hit by excess energy, rodents often store excessive fat in both adipose tissue and liver, leading to obesity and fatty liver; in contrast, the tree shrew accumulates fat in liver exlusively, not in adipose tissue, displaying non-obese fatty liver.

Theoretically, in rodent models, high energy intake often results in fat distribution to both adipose tissues, especially epididymal adipose tissue and visceral adipose tissue, and to the liver, thus leading to obesity and fatty liver. However, in tree shrews, fat distribution to adipose tissues is shut down by uncharacterized mechanisms; while the liver of tree shrew is quite sensitive to accumulating fat under excessive energy conditions (Fig. 7). The discrepancy between the rodent and tree shrew model of NAFLD may facilitate researchers to select an appropriate animal model for the research of human NAFLD. In brief, we have successfully established tree shrew models of human NAFLD by the induction of high-energy diets, in which they display dyslipidemia and hepatic steatosis, but not hyperglycemia and obesity. Thus, tree shrews can be used as a completely novel animal model to study human non-obese NAFLD.

## MATERIALS AND METHODS

### Animals and experimental design

All tested tree shrews were males of around one year of age raised in the Kunming Institute of Zoology (KIZ), Chinese Academy of Sciences (CAS). All animal experiments were carried out according to the guidelines approved by the Animal Ethics Committee of the Kunming Institute of Zoology, Chinese Academy of Science. Animals were housed one animal per cage at room temperature maintained at 21±2°C, humidity at 50-70%, with natural lighting and free access to food and water.

The diets and processes of the four approaches were listed in Table S1 and S2. At the sampling time point, animals were fasted overnight (∼14 h) and then euthanized by ethyl ether anesthesia. Blood and liver samples were harvested, livers of tree shrews were fixed in 10% formalin or snap frozen in liquid nitrogen and stored at −80°C for further analyses.

### Plasma biochemical parameters

Femoral vein blood of each tree shrew (0.5 ml) was taken after fasting 14 h, collected into the EDTAK_2_-containing glass tubes (Shandong Aosaite Medical Devices Co’ LTD, Heze, Shandong, China), and then centrifuged at 664 ***g*** (3000 rpm) for 5 min at room temperature. The plasma levels of aspartate aminotransferase (AST), alanine aminotransferase (ALT), triglycerides (TG), total cholesterol (TC), low density lipoprotein-cholesterol (LDL-c), high density lipoprotein-cholesterol (HDL-c), fasting blood glucose (FBG) and glycated haemoglobin A1c (HbA1c) were assayed by an automatic blood biochemistry analyzer (Abbott CI16200, Chicago, IL, USA) at the First People's Hospital of Yunnan Province, Kunming, China.

### Hepatic histology

From the live sample, 5 μm-thick sections of formalin-fixed and paraffin-embedded livers were processed for hematoxylin-eosin (H&E) staining. Liver sections were then visualized and photographed using a light microscope (Olympus BX53, Kanngawa, Japan).

### Analysis of hepatic gene expression by real-time quantitative PCR (QPCR)

The extraction of total RNA from liver tissues and the performance of real time quantitative PCR (QPCR) followed as we described previously (Zhang et al., 2015b).

### Statistical analysis

Data were presented as mean±s.e.m. Statistical analysis was performed using one-way analysis of variance (ANOVA) followed by LSD multiple comparison tests by SPSS20.0 (IBM SPSS Statistics, Armonk, NY, USA). All figures were made using GraphPad Prism 5 (GraphPad Software, La Jolla, CA, USA).
